# CCR2 recruits monocytes to the lung, while CX3CR1 modulates positioning of CD11c^pos^ cells in the lymph node during pulmonary tuberculosis

**DOI:** 10.1128/mbio.01237-25

**Published:** 2025-06-11

**Authors:** Alexander Mohapatra, Brian A. Norris, Zachary Howard, Joel D. Ernst

**Affiliations:** 1 Division of Experimental Medicine, Department of Medicine, University of California, San Francisco, California, USA; 2 Division of Infectious Diseases, New York University School of Medicine 12296 , New York, New York, USA; Weill Cornell Medicine, New York, New York, USA

**Keywords:** *Mycobacterium tuberculosis*, monocytes, immunity, cell trafficking

## Abstract

Infection by *Mycobacterium tuberculosis* (Mtb) continues to cause more than 1 million deaths annually, due to pathogen persistence in lung macrophages and dendritic cells derived from blood monocytes. While the accumulation of monocyte-derived cells in the Mtb-infected lung partially depends on the chemokine receptor CCR2, the other chemoattractant receptors regulating trafficking remain undefined. We used mice expressing knock-in/knockout reporter alleles of *Ccr2* and *Cx3cr1* to interrogate their expression and function in monocyte-derived populations of the lungs and draining mediastinal lymph nodes during Mtb infection. CCR2 and CX3CR1 expression varied across monocyte-derived subsets stratified by cell surface Ly6C expression in both organs. We found that the expression of CCR2 predicted the dependence of monocyte-derived cells on the receptor for lung and lymph node accumulation. CCR2-deficient mice were also observed to have worsened lung and lymph node Mtb burden. While CX3CR1 deficiency, alone or in combination with CCR2 deficiency, did not affect cell frequencies or lung Mtb control, its absence was associated with altered positioning of monocyte-derived dendritic cells in mediastinal lymph nodes. We found that the combined loss of *Ccr2* and *Cx3cr1* also worsened Mtb control in the mediastinal lymph node, suggesting a rationale for the persistent expression of CX3CR1 among monocyte-derived cells in pulmonary tuberculosis.

**IMPORTANCE:**

*Mycobacterium tuberculosis* (Mtb) is the respiratory pathogen responsible for the deadliest infectious disease worldwide. Susceptible humans exhibit ineffective immune responses, in which infected phagocytes are not able to eliminate the pathogen. Since recruited monocyte-derived cells serve as reservoirs for persistent infection, understanding how these phagocytes accumulate in the lung and why they are unable to eliminate Mtb can inform the development of therapies that can synergize with antimicrobials to achieve faster and more durable Mtb elimination. Monocyte-derived cells express the chemokine receptors CCR2 and CX3CR1, but the role of the latter in Mtb infection remains poorly defined. The significance of our study is in elucidating the roles of these receptors in the trafficking of monocyte-derived cells in the infected lung and mediastinal lymph node. These data shed light on the host response in tuberculosis and other pulmonary infections.

## INTRODUCTION

Tuberculosis remains one of the deadliest infectious diseases worldwide, causing more than 1 million deaths annually (1). Humans who progress to disease following inhalation of *Mycobacterium tuberculosis* (Mtb) exhibit ineffective immune responses, in which infected phagocytes are not able to eliminate the mycobacteria (2). Since recruited monocyte-derived cells serve as reservoirs for persistent infection (3–6), understanding how these phagocytes accumulate in the lung and why they are unable to eliminate Mtb can inform the development of therapies that can synergize with antimicrobials to achieve faster and more durable Mtb elimination.

Following phagocytosis by alveolar macrophages (AMs) (7–9), Mtb establishes a durable niche in the lung parenchyma within phagocytes derived from blood monocytes (3–6, 10). Monocyte-derived macrophages become the dominant infected cells within the lungs of mice, while monocyte-derived dendritic cells (DCs) traffic Mtb to draining lymph nodes (LNs) for T cell activation (9, 11–14). However, T cells do not effectively eliminate Mtb from monocyte-derived lung macrophages (4, 5, 15–17). Moreover, blood monocytes are continuously recruited to the infected lung (18, 19), creating a renewable reservoir of Mtb. Therefore, a key question is how monocytes traffic to the lungs and LNs during Mtb infection.

Two phenotypes of blood monocytes, classical and non-classical, are defined by the expression of the chemokine receptors CCR2 and CX3CR1, respectively, suggesting their importance to monocyte biology (20). CCR2 deficiency impairs monocyte egress from the bone marrow and may affect recruitment to sites of inflammation (21–23). Cell surface components of Mtb (24) and type I interferons (25) induce the CCR2 ligand CCL2 in Mtb infection, enhancing monocyte recruitment to the lung. Studies of infected, CCR2-deficient mice demonstrate that the receptor is essential for maximal accumulation of monocyte-derived cells in the lungs (26, 27). Diminished monocyte recruitment is associated with impaired Mtb control, likely due to reduced activation of Mtb-specific T cells (13, 26–29). However, mononuclear phagocyte recruitment is not completely abrogated in the absence of CCR2, indicating that one or more other chemoattractant receptors contribute to this response to infection. Expression of the CX3CR1 ligand CX3CL1 has not been characterized in Mtb infection, but it is highly expressed in the human lung at baseline (30). CX3CR1^pos^ monocyte-derived cells have been observed to phagocytose *Mycobacterium bovis* bacille Calmette-Guérin during pulmonary infection (31). However, Mtb-infected *Cx3cr1^−/−^* mice exhibited similar mycobacterial control to that of wild-type mice (32), although phagocyte accumulation in the lungs was not reported in that study. Given that monocytes highly express CCR2 and CX3CR1 and that monocyte trafficking to the Mtb-infected lung is not completely abrogated in CCR2-deficient mice, we generated mice deficient in both CCR2 and CX3CR1 and used them to evaluate the accumulation of monocyte-derived cells and Mtb control in the lungs and mediastinal LNs (MLNs) following aerosol infection.

We used the knock-in/knockout *Ccr2^RFP/RFP^* (33) and *Cx3cr1^GFP/GFP^* (34) strains to breed double knockout (DKO) mice and double heterozygous mice, for tracking *Ccr2*- and *Cx3cr1*-expressing phagocytes during pulmonary Mtb infection. Mice that are heterozygous for these alleles exhibit normal migratory properties attributable to the remaining intact copy of each chemokine receptor gene and express the respective reporter. Mice that are homozygous at each targeted locus are null for the respective chemokine receptor. We observed variable receptor expression among monocyte-derived subsets and differential dependence on these receptors for the accumulation of these cells in the lung parenchyma and the MLN. While CCR2-deficient and DKO mice had similar impairment of lung Mtb control, compared with that in CCR2-replete mice, the combined absence of Ccr2 and Cx3cr1 was associated with altered positioning of monocyte-derived DCs in the MLN and with higher MLN Mtb burdens.

## RESULTS

### Variable expression of Ccr2 and Cx3cr1 by mononuclear phagocyte subsets in *M. tuberculosis*-infected mice

**Fig 1:**
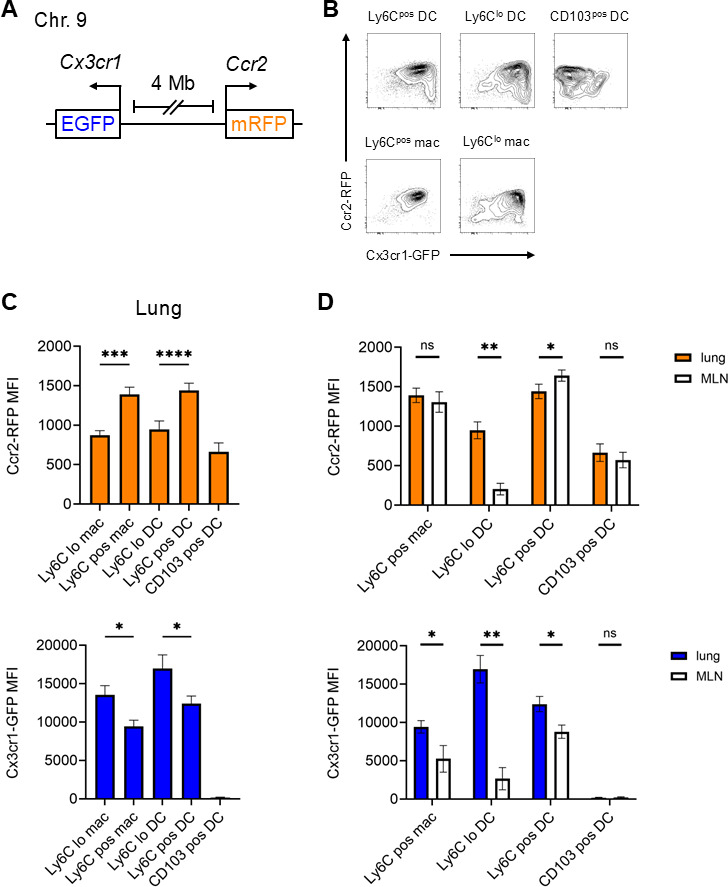
A dual *Ccr2*-*Cx3cr1* reporter mouse demonstrates varied receptor expression by cell type and location. (**A**) Schematic of the dual reporter genes on chromosome 9. (**B through D**) *Ccr2^RFP/+^*; *Cx3cr1^GFP/+^* mice were infected with a target dose of 100 CFUs of Mtb, then euthanized at 4 weeks post-infection for analysis of lung and MLN cells by flow cytometry (gating in Fig. S1). (**B**) Representative contour plots of the indicated populations from the lungs of infected mice (“mac”: monocyte-derived macrophage). (**C**) MFIs of RFP (top) and GFP (bottom) in lung cells. (**D**) Comparison of RFP (top) and GFP (bottom) MFIs among equivalent lung and MLN populations (Ly6C^lo^ macrophages were not observed in significant numbers in the MLN). Data in panels **B through D** are from 1 of the 2 experiments with similar results, each conducted with 4 mice per genotype. All results are presented as arithmetic mean + SD. *, *P* < 0.05; **, *P* < 0.005; ***, *P* < 0.0005; ****, *P* < 0.00005; ns, not significant; by one-way analysis of variance (ANOVA) with multiple comparisons (**C**) or *t* test (**D**).

Since *Ccr2* and *Cx3cr1* are genetically linked on *M. musculus* chromosome 9, studying the effects of dual receptor deficiency on phagocyte trafficking required mating of F1 littermates until a crossing-over event generated a dual reporter allele (Fig. 1A). To study the expression of these receptors in specific cell subsets, we infected *Ccr2^RFP/+^*; *Cx3cr1^GFP/+^* (denoted “Het” hereafter) mice with Mtb and used flow cytometry to analyze cells isolated from lungs and MLNs 4 weeks post-infection (Fig. 1B). We measured red fluorescent protein (RFP) and green fluorescent protein (GFP) median fluorescence intensity (MFI), as reflective of receptor gene expression, in monocyte-derived macrophages, monocyte-derived DCs, AMs, and neutrophils (gating in Fig. S1). Expression was also assessed in CD103^pos^ conventional DCs, which are rarely infected in the lung but present Mtb-derived antigens in MLNs (13, 14).

We assessed RFP and GFP expression by organ and cell type (Fig. 1B). Intravascular (IV) anti-CD45 antibody injection prior to euthanasia was used to distinguish intravascular populations (CD45-IV^pos^), including Ly6C^hi^ CCR2^hi^ CX3CR1^lo^ classical and Ly6C^lo^ CCR2^lo^ CX3CR1^hi^ non-classical monocytes, from those in the lung parenchyma (CD45-IV^neg^). In Mtb-infected mice, we have previously used adoptive transfer of monocytes to determine that parenchymal populations are derived from circulating classical monocytes, which transition from Ly6C^pos^ to Ly6C^lo^ over time (19) and assume macrophage-like or DC-like phenotypes in the lung (12). We predicted that these monocyte-derived cells would have similar chemokine receptor expression kinetics as do blood monocytes, which lose CCR2 and Ly6C expression and acquire CX3CR1 expression when transitioning from classical monocytes into non-classical monocytes (35). This prediction was correct, as Ly6C^lo^ cells had lower RFP (*Ccr2* reporter) and higher GFP (*Cx3cr1* reporter) expression than did Ly6C^pos^ populations (Fig. 1C). Parenchymal CD103^pos^ DCs did not express significant levels of GFP but had detectable RFP expression. AMs and neutrophils expressed minimal RFP or GFP (Fig. S2), as expected (21, 34, 36, 37).

We previously found that both Ly6C^pos^ and Ly6C^lo^ monocyte-derived populations migrate from the lung parenchyma to the MLN in Mtb-infected mice (19), so we predicted that expression of CCR2 and CX3CR1 would be similar between lung and MLN populations. While MLN Ly6C^pos^ macrophages and DCs resembled lung populations in RFP and GFP MFI, MLN Ly6C^lo^ DCs had significantly lower expression of both reporters than did equivalent cells in the lung (Fig. 1D). CD103^pos^ DCs in the MLN had expression of CCR2 and CX3CR1 comparable to that in lung cells. For all lung and MLN populations evaluated, RFP and GFP expression peaked at 4 weeks post-infection and were significantly decreased by 7 weeks (Fig. S3A and B).

### Lung and MLN phagocytes differ in their dependence on CCR2 and CX3CR1

**Fig 2:**
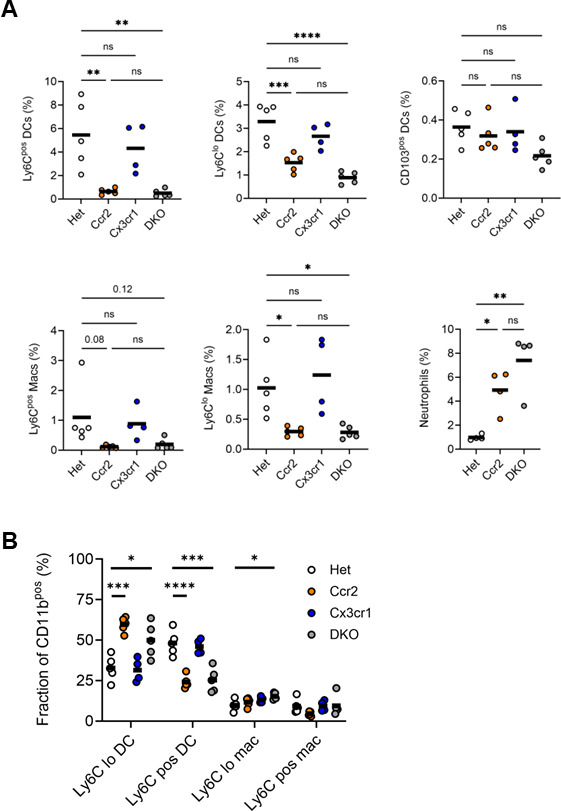
Phagocyte frequencies in the lungs of Mtb-infected CCR2-deficient, CX3CR1-deficient, and DKO mice. *Ccr2^RFP/+^*; *Cx3cr1^GFP/+^* (Het), *Ccr2^RFP/RFP^* (Ccr2), *Cx3cr1^GFP/GFP^* (Cx3cr1), and DKO mice were infected with ∼100 CFUs Mtb for 4 weeks, and lungs were harvested for analysis. (**A**) Frequencies of the indicated lung populations among live cells by flow cytometry (live cell gate defined in Fig. S1). (**B**) Proportions of the indicated populations among CD11b^pos^ cells (parent gate defined in Fig. S1). Data are from 1 of the 2 experiments with similar results, each conducted with four mice per genotype. The horizontal bar represents the arithmetic mean in all graphs. Significance assessed by one-way ANOVA with multiple comparisons.

Our chemokine receptor expression data suggested that Ly6C^lo^ monocyte-derived cells might utilize CX3CR1 for recruitment to the lung parenchyma, potentially explaining the partial trafficking defect observed with CCR2 deficiency alone. To test this hypothesis, we measured the accumulation of monocyte-derived cells in the lungs and MLNs of Mtb-infected mice deficient in CCR2, CX3CR1, or both receptors. We confirmed that Mtb-infected Het mice had a similar accumulation of lung phagocyte populations as did wild-type (*Ccr2^+/+^*; *Cx3cr1^+/+^*) mice (Fig. S4A) and could be used as controls. In the lung at 4 weeks post-infection, accumulation of Ly6C^pos^ macrophages and DCs declined by more than 80% in CCR2-deficient (*Ccr2^RFP/RFP^*) and DKO mice, while CX3CR1-deficient (*Cx3cr1^GFP/GFP^*) mice had preserved trafficking relative to Het mice (Fig. 2A; Fig. S5A). Accumulation of Ly6C^lo^ macrophages and DCs, however, was less dependent on CCR2, although the additional loss of CX3CR1 did not further reduce the frequencies of these populations. Whereas Ly6C^pos^ DCs were the most prevalent monocyte-derived subset in Het and CX3CR1-deficient mice, the residual monocyte-derived cells in CCR2-deficient and DKO mice were Ly6C^lo^ DCs (Fig. 2B). Overall, our data suggest that while CCR2 expression predicts accumulation of monocyte-derived cells in the lungs of Mtb-infected mice, CX3CR1 expression does not. Conversely, despite expressing detectable RFP (Fig. 1C), accumulation of CD103^pos^ DCs was not dependent on CCR2 (Fig. 2A). The absence of CCR2 was also associated with increased parenchymal neutrophils. Given that neutrophils did not express CCR2, as indicated by RFP expression (Fig. S2) and in the literature (21), this is likely an indirect effect potentially due to higher mycobacterial burdens (26, 27, 29).

**Fig 3:**
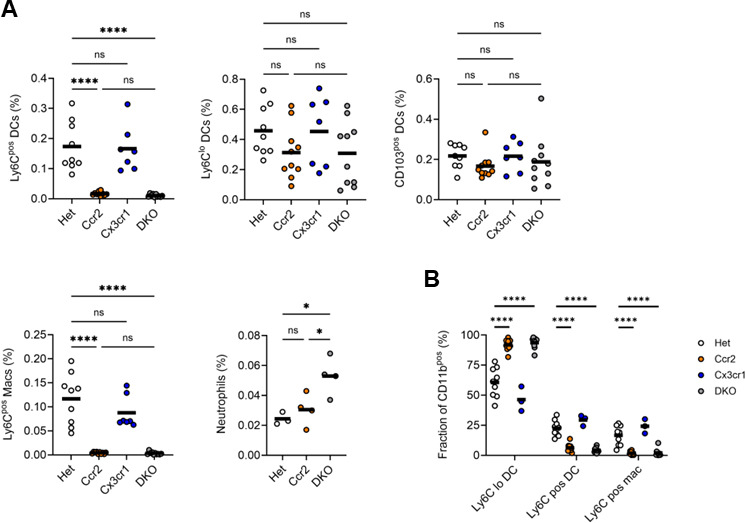
Phagocyte frequencies in the MLNs of Mtb-infected CCR2-deficient, CX3CR1-deficient, and DKO mice. Mice were infected, as in Fig. 2, and MLNs were harvested for analysis. (**A**) Frequencies of the indicated MLN populations among live cells by flow cytometry (live cell gate defined in Fig. S1). (**B**) Proportions of the indicated populations among CD11b^pos^ cells (parent gate defined in Fig. S1). Data are from 1 of the 2 experiments with similar results, each conducted with four mice per genotype. The horizontal bar represents the arithmetic mean in all graphs. Significance assessed by one-way ANOVA with multiple comparisons.

The significantly lower CCR2 and CX3CR1 expression in MLN Ly6C^lo^ DCs, relative to lung Ly6C^lo^ DCs, suggested that trafficking of this population to the MLN does not depend on these receptors. This was confirmed, as the accumulation of Ly6C^lo^ and CD103^pos^ DCs was similar in the MLNs of infected Het, single, and double knockout mice (Fig. 3A; Fig. S5B). MLN Ly6C^pos^ macrophages and DCs, which expressed the CCR2 reporter, were nearly absent in CCR2-deficient and DKO mice. Consequently, Ly6C^lo^ DCs were the dominant monocyte-derived population in CCR2-deficient and DKO MLNs (Fig. 3B). Neutrophils were the only MLN phagocyte significantly affected by CX3CR1 deficiency. The absence of both CCR2 and CX3CR1 was associated with an increase in neutrophil frequency (Fig. 3A), again presumed to be an indirect effect (Fig. S2).

### Mtb-infected cells exhibit altered positioning in MLNs of DKO mice

**Fig 4:**
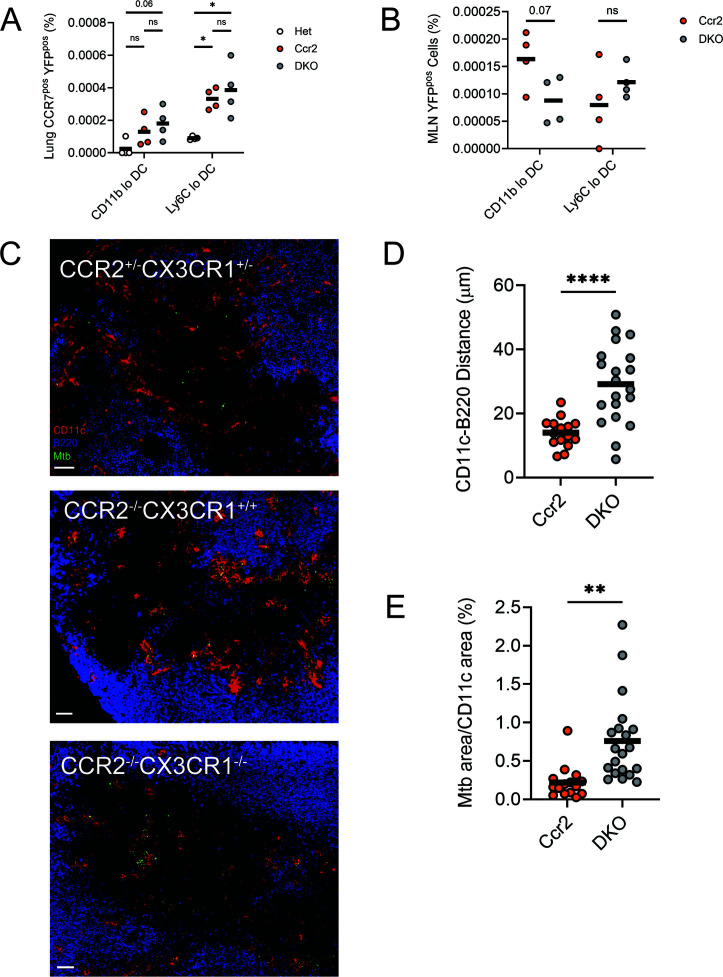
Mtb-infected cells exhibit altered positioning in the MLNs of CCR2–CX3CR1 DKO mice. Lungs and MLNs were harvested from mice infected with ∼100 CFUs of YFP-expressing Mtb for 4 weeks. (**A**) Frequency of CCR7^pos^ YFP^pos^ cells of the indicated subsets among total lung cells. (**B**) Frequency of YFP^pos^ cells of the indicated subsets among total MLN cells. Data in panels **A and B** are from 1 of the 2 experiments with similar results, each conducted with four mice per genotype. (**C**) Representative immunofluorescence staining of non-follicular zones in MLN sections from Het, Ccr2, or DKO mice. Scale bar is 70 µm. (**D**) For each CD11c^pos^ cell in non-follicular zones, the distance to the closest B220^pos^ cell was measured. (**E**) Mtb-positive staining area was measured as a percentage of the total CD11c-positive staining area in non-follicular zones. Graphs in panels **D and E** show the average distance by non-follicular zone from sections of the indicated genotype and are compiled from three experiments. Horizontal bar represents the arithmetic mean in all graphs. Significance assessed by one-way ANOVA with multiple comparisons (**A**) or *t* test (**B, D, and E**).

To further examine the potential effects of chemokine receptor deficiency on the recruitment and function of mononuclear cells in the MLN, we infected CCR2-deficient and DKO mice with an Mtb strain expressing yellow fluorescent protein (YFP) (38). This permitted the identification of infected phagocytes in the lungs and MLNs with simultaneous detection of the GFP and RFP reporters (Fig. S1). To measure the transport of Mtb to the MLN by infected lung cells, we quantitated the frequency of CCR7^pos^ phagocytes at 4 weeks post-infection and observed that CCR2 deficiency was associated with more CCR7^pos^ YFP^pos^ Ly6C^lo^ DCs in the lungs (Fig. 4A). However, additional loss of CX3CR1 did not further increase the frequency of these cells. Some CCR7^pos^ YFP^pos^ CD11b^lo^ conventional DCs (both CD103^lo^ and CD103^pos^; not shown) were observed in the lungs, but these were less prevalent than CD11b^pos^ Ly6C^lo^ monocyte-derived DCs and were not affected by the absence of CCR2 or CX3CR1. We next quantified Mtb-infected cells in the MLN and observed that YFP^pos^ DCs could not be detected in Het mice (not shown). However, YFP^pos^ CD11b^lo^ and Ly6C^lo^ DCs were observed in the MLNs of CCR2-deficient and DKO mice. Animals lacking both CCR2 and CX3CR1 had fewer YFP^pos^ CD11b^lo^ DCs but a similar frequency of YFP^pos^ Ly6C^lo^ DCs, compared with mice deficient in CCR2 alone (Fig. 4B).

Although the frequency of Ly6C^lo^ DCs in the MLNs of infected mice was not affected by deficiency of CCR2 or CX3CR1 (Fig. 3A), we asked whether Ly6C^lo^ DCs in DKO mice had altered positioning within MLNs. We used immunofluorescence microscopy of MLN sections to identify CD11c^pos^ cells in non-follicular regions (defined as B220^neg^; Fig. 4C), including T cell zones (Fig. S6A). The average distance of CD11c^pos^ cells to a follicle border (the nearest B220^pos^ cell) in these regions was measured (Fig. 4D). CD11c^pos^ cells tended to be farther away from a B220^pos^ area in DKO mice than they were in CCR2-deficient mice. Positioning of CD11c^pos^ cells was not directly compared between Het mice and either CCR2-deficient or DKO mice because of the disparity in Ly6C^pos^ DCs (Fig. 3A) and Mtb burden (see Fig. 6B). We also quantified YFP fluorescence and observed that CD11c^pos^ cells in non-follicular regions of DKO MLNs had more Mtb burden than did cells in equivalent regions of CCR2-deficient mice (Fig. 4E). Since we observed more neutrophils in the MLNs of infected DKO mice (Fig. 3A), we also analyzed co-localization of Ly6G and YFP in MLN sections and found few infected neutrophils (Fig. S6B).

**Fig 5:**
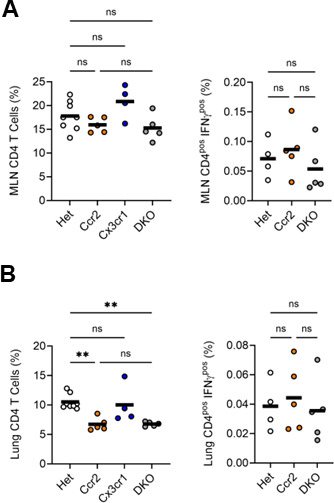
Accumulation and polarization of CD4 T cells in MLNs and lungs of CCR2-deficient, CX3CR1-deficient, and DKO mice. Frequencies of total and IFNγ^pos^ CD4 T cells in the MLN (**A**) and lung (**B**) 4 weeks after infection with ∼100 CFUs of Mtb. Data are from 1 of the 2 experiments with similar results, each conducted with four mice per genotype. The horizontal bar represents the arithmetic mean in all graphs. Significance assessed by one-way ANOVA with multiple comparisons.

We then assessed how the change in MLN DC populations induced by CCR2 deficiency affected the accumulation and polarization of CD4 T cells. In the MLN, the frequency of total CD4 T cells was not affected by the absence of CCR2 or CX3CR1 (Fig. 5A). In contrast, CD4 T cell frequency in the lung parenchyma was reduced by 35% in CCR2-deficient mice when compared with Het mice (Fig. 5B). There was no difference in lung CD4 T cell frequency between mice lacking CCR2 alone and those lacking both receptors. While CCR2 deficiency was associated with reduced overall frequency of CD4 T cells in the lungs, the frequency of IFNγ^pos^ CD4 T cells in both the MLN (Fig. 5A) and the lung (Fig. 5B) was not affected by single or dual deficiency of CCR2 or CX3CR1.

### Combined deficiency of CCR2 and CX3CR1 worsens Mtb control in the MLN but not the lung compared with CCR2 deficiency alone

**Fig 6:**
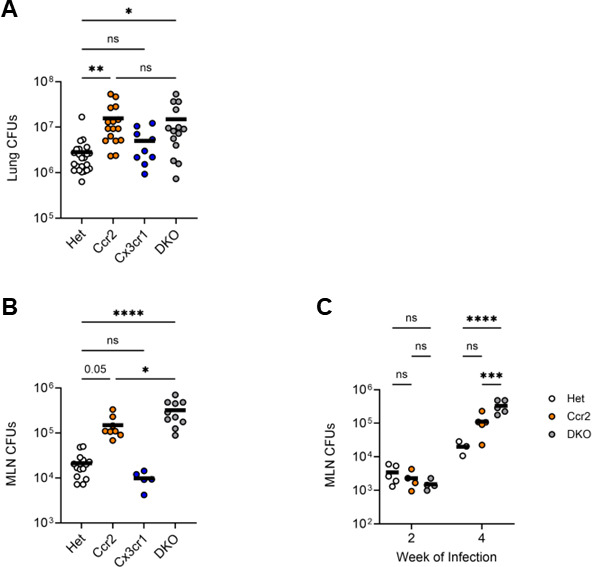
DKO mice have a greater mycobacterial burden in MLNs than CCR2-deficient mice. Mice were infected for 2 or 4 weeks with ∼100 CFUs of Mtb, then lungs and MLNs were harvested. (**A**) Quantitation of Mtb bacilli in the lungs of infected mice by colony formation (CFUs) on agar. Data are compiled from four experiments. (**B**) Quantitation of Mtb bacilli in the MLNs of mice infected for 4 weeks. Data are compiled from three experiments. (**C**) MLN CFUs after 2 and 4 weeks of infection. Data are from 1 of the 2 experiments with similar results, each conducted with three mice per genotype. The horizontal bar represents the arithmetic mean in all graphs. Significance assessed by one-way (**A and B**) or two-way (**C**) ANOVA with multiple comparisons.

We next quantified viable Mtb bacilli in the lungs of Het, Ccr2 and Cx3cr1 single knockouts, and DKO mice at 4 weeks post-infection. We found significantly more Mtb CFUs in the lungs of CCR2-deficient and DKO mice (Fig. 6A), compared to those in Het mice (which had similar Mtb burdens as wild-type *Ccr2^+/+^*; *Cx3cr1^+/+^* mice; Fig. S4C). Mtb CFUs in mice lacking both receptors did not differ from those in mice lacking CCR2 alone. In contrast, mycobacterial burdens did not differ in the lungs of CX3CR1-deficient mice compared with those in Het mice. Together, these results with CCR2-deficient mice are similar to those in prior studies (26, 27, 29) and reveal that deficiency of CX3CR1 does not impact lung bacterial burdens, even when combined with CCR2 deficiency.

In contrast to the findings in the lungs, in the MLNs of mice infected with Mtb for 4 weeks, deficiency of both CCR2 and CX3CR1 was associated with higher mycobacterial burdens compared to those in single knockouts (Fig. 6B). As observed in the lungs, CCR2-deficient mice had more bacilli in MLNs than did CX3CR1-deficient mice, which were similar to those in Het mice. To determine whether the difference in bacilli observed in the MLNs at 4 weeks post-infection was present at an earlier phase of the infection, we quantified Mtb bacilli at 2 weeks post-infection. MLN Mtb burden was lower than at 4 weeks post-infection, as expected, but was not affected by CCR2 or CX3CR1 deficiency at the earlier time point (Fig. 6C).

## DISCUSSION

We generated and used dual reporter and knockout mice to define the expression and roles of the chemokine receptors CCR2 and CX3CR1 in lung mononuclear phagocytes during Mtb infection. Distinct monocyte-derived cell subsets were observed to have specific patterns of *Ccr2* and *Cx3cr1* expression related to cell surface Ly6C and localization within the lung parenchyma or the MLN. We confirmed that CCR2-deficient mice have significantly reduced accumulation of Ly6C^pos^ monocyte-derived cells in the lung and MLN, with higher lung and MLN Mtb burden. CX3CR1 deficiency, alone or in combination with CCR2 deficiency, did not affect monocyte-derived populations or Mtb control in the lung but was associated with higher mycobacterial burdens in the MLN and altered positioning of monocyte-derived Ly6C^lo^ DCs.

Dual reporter mice revealed dynamic regulation of *Ccr2* and *Cx3cr1* in monocyte-derived cells following extravasation into the Mtb-infected lung. Our finding—that Ly6C expression directly correlates with that of *Ccr2* and inversely correlates with *Cx3cr1* among lung monocyte-derived cells, independent of whether these cells adopted a macrophage-like or a DC-like phenotype—mimics the distinction between Ly6C^hi^ CCR2^hi^ CX3CR1^lo^ and Ly6C^lo^ CCR2^lo^ CX3CR1^hi^ monocytes in the blood (20). The cells we profiled are extravascular, and we have previously shown that Ly6C^hi^ bone marrow monocytes differentiate into Ly6C^lo^ cells over time within the lung parenchyma (19), suggesting that they are not derived from blood Ly6C^lo^ cells. Therefore, our results are consistent with a shared program between blood and lung monocytes, whereby these cells repress *Ccr2* and induce *Cx3cr1* expression, possibly via induction of *N4ra1* (39). Similar receptor expression kinetics have been observed among monocyte-derived cells in the peritoneum (34), skin (40, 41), skeletal muscle (42), myocardium (43), gut (44), and liver (45). Our finding that CCR2 deficiency results in a greater proportion of Ly6C^lo^ DCs in the infected lung implies that these cells are a more stable, terminal population, occupying a niche that still fills, but at a slower rate, when accumulation of Ly6C^pos^ monocytes is reduced. Accumulation of a long-lived monocyte-derived population may underlie our observation that both *Ccr2* and *Cx3cr1* expression appear to decline in all phagocytes analyzed as the infection progresses, although we cannot exclude that systemic changes in monocyte receptor expression also occur with prolonged infection.

We and others have previously shown that CCR2 (13, 26, 28) and CCR7 (10, 11, 19, 46) contribute to the accumulation of monocyte-derived DCs and to T cell activation in the MLNs of Mtb-infected mice. Our data on *Ccr2* expression and its role in the trafficking of monocyte-derived populations to the lung and MLN suggest a model in which CCR2 facilitates monocyte accumulation in the infected lung, consistent with other studies (21–23), while CCR7 regulates MLN entry. Since our data and those from other studies (26–29) suggest that monocyte trafficking to the Mtb-infected lung is delayed, rather than completely abrogated, in CCR2-deficient mice, other chemokine receptors must contribute to monocyte trafficking. However, we also do not observe a role for CX3CR1 in the accumulation of monocyte-derived cells in the Mtb-infected lung or MLN, despite detectable expression. The LN result agrees with other data showing that CX3CR1^pos^ monocyte-derived DCs traffic to LN T cell zones in the spleen (34) and the intestines (47) in a CX3CR1-independent manner.

Our observation that DKO mice have increased MLN Mtb bacilli compared to single knockouts and controls reveals a novel role for CX3CR1. We (10, 14, 17, 19) and others (13) have previously shown that viable Mtb is transported to the MLN from the lung 1–2 weeks post-infection via monocyte-derived cells, which subsequently transfer Mtb antigens, but not live mycobacteria, to conventional DCs for T cell priming. Since we did not observe an increase in MLN Mtb burden in DKO mice until 4 weeks post-infection and since neither receptor is required for MLN entry, the worsened Mtb control is unlikely to be explained by trafficking of more infected cells to the MLN from the lung. Rather, we found that the altered positioning of Ly6C^lo^ DCs away from follicular regions, due to CX3CR1 deficiency, was associated with Mtb survival within these cells. This phenotype is likely apparent only in DKO mice, and at a later stage of infection, because of two effects. First, significantly reduced trafficking of Ly6C^pos^ DCs to the MLN impairs T cell activation, resulting in higher lung Mtb burden and inflammation (as indicated by increased neutrophil accumulation) over time. Second, delayed accumulation of Ly6C^lo^ DCs in the lung eventually provides an alternative path for Mtb to reach the MLN. Conversely, Cx3cr1 single-knockout mice have an earlier influx of Ly6C^pos^ DCs to the MLN, resulting in antigen transfer to conventional DCs that do not express the receptor and normal activation of Mtb-specific T cells.

While CX3CR1^pos^ monocyte-derived macrophages in the uninfected lung have been associated with bronchiole-associated nerves (48), the tissue distribution of the chemokine itself has not been described, so the functional role of the receptor for these macrophages remains unclear. Similarly, the expression of CX3CR1 ligands has not been defined in MLNs. Our data suggest that a chemokine gradient does exist in Mtb infection and directs Ly6C^lo^ DCs to the T cell zone-B cell follicle border. However, we were unable to visualize CX3CL1 using commercially available reagents. In the adaptive immune response to Mtb, this gradient may not be relevant because we have previously shown that infected Ly6C^lo^ DCs poorly present antigen to T cells (14, 17). It is unclear why altered positioning of Ly6C^lo^ DCs affects Mtb survival or antigen export to conventional DCs. Our data also establish that CX3CR1 does not act redundantly with CCR2 to traffic monocytes to the infected lung.

Several groups have studied Mtb infection of Ccr2 (27, 29) or Cx3cr1 (32) single-knockout mice to elucidate how monocyte trafficking establishes the Mtb niche in the lung. Our data are consistent with these prior studies but further extend them by demonstrating a unique role for CX3CR1 in the MLN that may explain its coordinated regulation with CCR2 in monocyte-derived cells at sites of inflammation. Given the distinct functions of CX3CR1 and CCR2 in monocyte biology, the dual knock-in/knockout reporter strain used in this study may be a helpful tool to characterize these cells in a variety of inflammatory diseases.

## MATERIALS AND METHODS

### Mice

C57BL/6 mice (8–12 weeks old) were obtained from Jackson Laboratory. Mice infected with Mtb were housed in the Animal Biosafety Level 3 facility. Ccr2-RFP and Cx3cr1-GFP mice were purchased from The Jackson Laboratory, then bred to generate *Ccr2^RFP/+^*; *Cx3cr1^GFP/+^* and *Ccr2^RFP/RFP^*; *Cx3cr1^GFP/GFP^* mice. All animal protocols used here were approved by the New York University School of Medicine and the University of California, San Francisco, Animal Care and Use Committees.

### Mycobacterial strains, growth, and aerosol infection

Mtb H37Rv transformed with pMV261 containing the gene expressing superfolder yellow fluorescent protein was obtained from the laboratory of Christopher Sassetti (38). Mycobacteria were grown in Middlebrook 7H9 medium (BD) supplemented with 10% (vol/vol) ADC (albumin, dextrose, and catalase), 0.05% Tween 80, 0.2% glycerol, and 110 µg/mL hygromycin. Mice were infected with H37Rv or H37Rv::pMV261-sfYFP via aerosol using an inhalation exposure unit from Glas-Col, as previously described (4, 19). Mid-log cultures were centrifuged at 800 × *g* to pellet clumps. Clump-free cultures were then diluted, and 5 mL inoculum was added to the nebulizer. The target dose was 100 CFUs/mouse. Infection dose was determined by plating lung homogenates 24 hours post-infection on 7H11 agar plates and enumerating CFUs after 3 weeks of incubation at 37°C.

### Tissue harvests and processing for flow cytometry

Mice were anesthetized by inhalation of isoflurane and then retro-orbitally injected with 1 µg of anti-CD45 antibody (clone 30-F11) in 150 µL of phosphate-buffered saline (PBS). Three minutes after antibody injection, mice were euthanized by CO_2_ inhalation and cervical dislocation. Lungs and MLNs were collected and placed in Hanks’ balanced salt solution (HBSS) containing 50 µg/mL Liberase (Sigma) and 30 µg/mL DNase I (Sigma) or RPMI-1640 with 5% heat-inactivated fetal bovine serum (HI-FBS) (vol/vol), respectively. Lymph nodes were mashed through 70 µm cell strainers. Lungs were incubated at 37°C for 30 min, then processed with a gentleMACS dissociator (Miltenyi) and mashed through 70 µm strainers. Aliquots for plating on 7H11 agar for CFU enumeration were taken prior to centrifugation. Following centrifugation, lung cell suspensions were dissolved in ammonium-chloride-potassium (ACK) lysis buffer (Gibco) for red blood cell lysis. Lung and MLN single-cell suspensions were washed and resuspended in PBS with 3% HI-FBS (vol/vol) and 2 mM EDTA (FACS buffer).

For staining of surface antigens, cells were washed with PBS, then stained with viability dye Live-or-Dye 750/777 (Biotium) and anti-CD16/32 (clone 2.4G2; Fc receptor blockade) for 15 min at 4°C. Cells were then washed and resuspended in FACS buffer with fluorochrome-conjugated anti-CCR7 antibody (clone 4B12) for 30 min at 37°C. After washing, cells were resuspended in FACS buffer with a cocktail of fluorochrome-conjugated antibodies against CD3 (17A2), CD4 (GK1.5), CD11b (M1/70), CD11c (N418), CD19 (6D5), CD90.2 (30-H12), CD103 (2E7), Ly6C (HK1.4), Ly6G (1A8), MHCII I-A and I-E (M5/114.15.2), NK1.1 (S17016D), and Siglec F (S17007L) for 25 min at 4°C. Cells were then washed with PBS, fixed with 4% paraformaldehyde (vol/vol) for 30 min at room temperature, and washed again in PBS.

For staining of intracellular IFNγ, cells were fixed with BD Cytofix/Cytoperm solution following staining of surface antigens, then washed in BD Perm/Wash buffer. After washing, cells were resuspended in BD Perm/Wash buffer with anti-IFNγ antibodies (XMG1.2) for 30 min at 4°C. Afterward, cells were washed in Perm/Wash buffer, then in PBS. Samples were acquired using an LSR II (BD) conventional flow cytometer or an Aurora spectral flow cytometer (Cytek Biosciences). See Fig. S1 for gating.

### Lymph node immunofluorescence

Mice were euthanized, as above, and intact MLNs were embedded in Optimal Cutting Temperature (OCT) Compound (Sakura), then rapidly frozen in 100% ethanol with dry ice. Blocks were cut into 10 µm sections by cryostat (Leica), and sections were transferred to slides and crosslinked using a CryoJane tape transfer system. Slides were fixed in 4% paraformaldehyde overnight, then stored in PBS at 4°C until immunofluorescence staining. For staining, sections were blocked with 10% goat serum for 30 min, then stained with antibodies against CD11c and B220 (clone RA3–6B2) for 1 hour. After washing, sections were stained with isotype-specific, fluorochrome-conjugated secondary antibodies, followed by 4′,6-diamidino-2-phenylindole (DAPI). Sections were mounted with VECTASHIELD Vibrance Antifade Medium (Vector Laboratories). Images were captured using the 20× objective of a Nikon Ti inverted microscope with a DS-Qi2 camera. Frames in an 8 × 8 grid were stitched together and deconvoluted using NIS Elements software (Nikon).

At least three MLN sections 50 µm apart were analyzed per mouse. For each MLN image, B220-negative regions were manually selected in ImageJ (National Institutes of Health). To quantitate distances between CD11c^pos^ cells and the borders of B220^neg^ regions (the edges of B220^pos^ follicles), images were processed with a Gaussian noise filter, then segmented via global intensity thresholding of the CD11c and B220 channels using the ImageJ plugin DiAna (49). For each CD11c^pos^ object, the distance to the closest B220^pos^ object was measured.

To quantitate Mtb associated with CD11c^pos^ cells in B220^neg^ regions, masks of the CD11c channel were generated using global intensity thresholds and a 10 µm border. After thresholding the Mtb channel in each region, the “Analyze Particles” function was used to measure Mtb channel area within the corresponding CD11c mask area.

### Data analysis

Flow cytometry data were analyzed using SpectroFlo 3.3 (Cytek) and FlowJo 10.10.0. Median fluorescence intensities for GFP and RFP were calculated after subtracting values recorded for equivalent populations from Mtb-infected *Ccr2^+/+^*; *Cx3cr1^+/+^* mice. GraphPad Prism 10.4.1 (GraphPad) was used for graphical presentation and statistical analysis.

## SUPPLEMENTAL MATERIAL

Supplemental Figures mbio.01237-25-s0001.pdf Figures S1–S6.

ASM does not own the copyrights to Supplemental Material that may be linked to, or accessed through, an article. The authors have granted ASM a non-exclusive, world-wide license to publish the Supplemental Material files. Please contact the corresponding author directly for reuse.
