# Supplementary material for: CCR2 recruits monocytes to the lung, while CX3CR1 modulates positioning of CD11cpos cells in the lymph node during pulmonary tuberculosis
Source: mBio. 2025 Jun 11;16(7):e01237-25. doi: 10.1128/mbio.01237-25 (PMC12239560; doi:10.1128/mbio.01237-25)
Supplement: Supplemental Figures — Figures S1–S6. [file mbio.01237-25-s0001.pdf]

Supplemental Figure 1

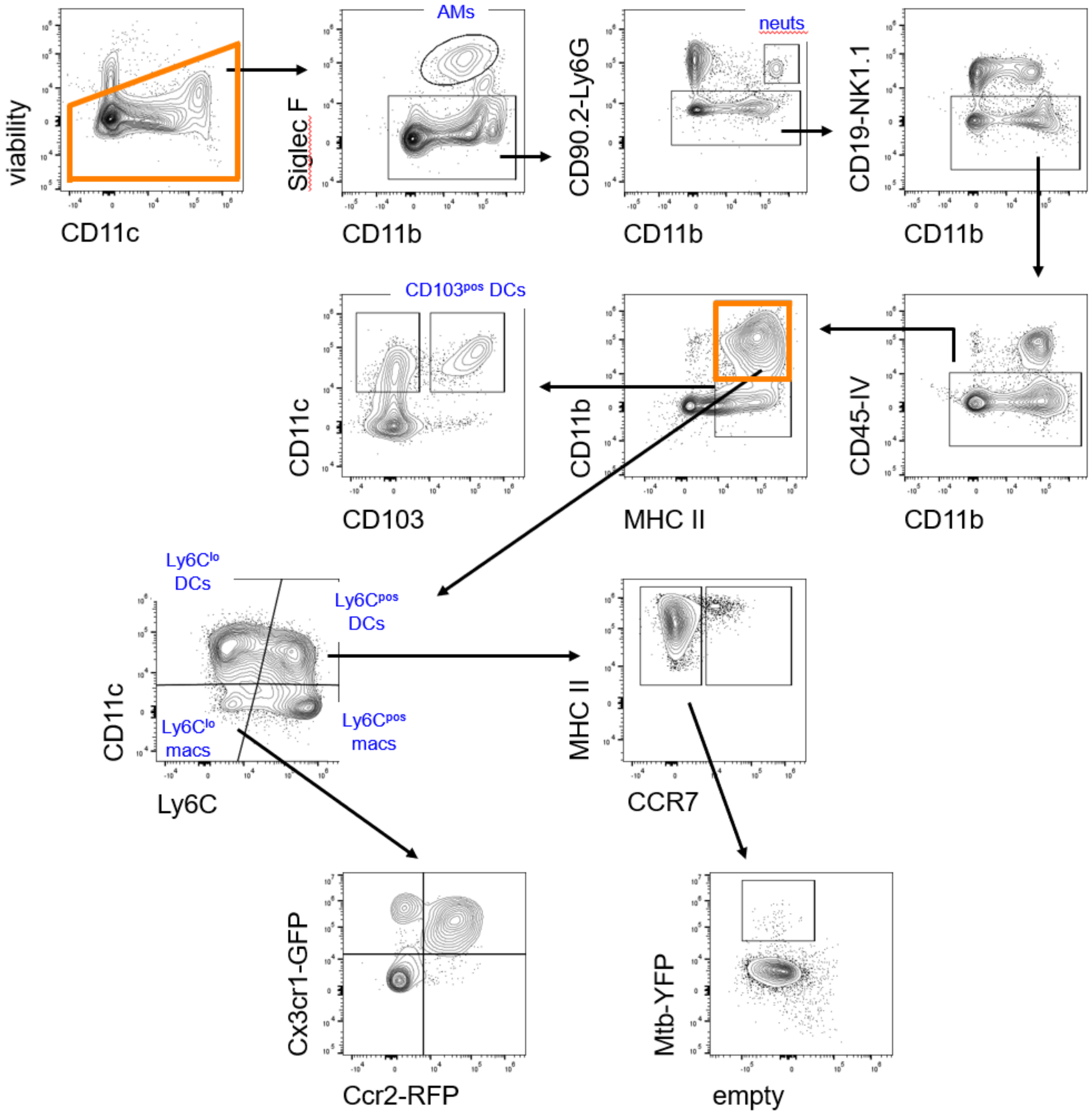

Supplemental Figure 1: Gating strategy for lung and MLN populations. Representative contour plots from the lungs of Mtb-infected mice (“neuts”: neutrophils). Gating for MLN samples was similar. Orange gates were used for frequency calculations among total live cells (Fig. 2A, 3A) or CD11b^{pos} cells (Fig. 2B, 3B). For analysis of T cells, CD3^{pos} CD4^{pos} events were gated from live cells (not shown).

Supplemental Figure 2

A

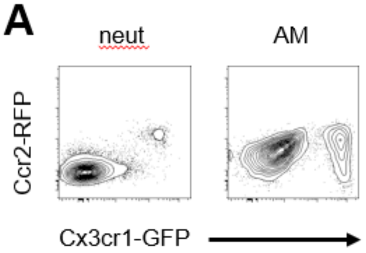

B

Lung

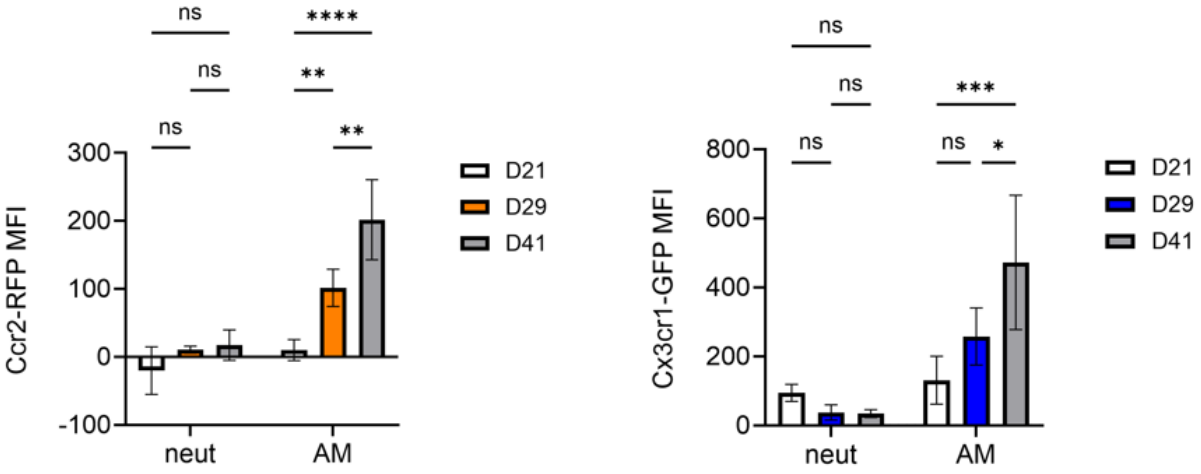

C

MLN neutrophils

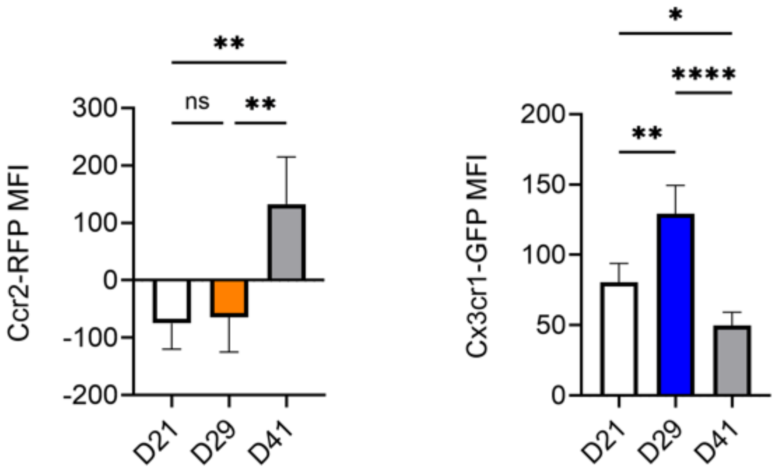

Supplemental Figure 2: *Ccr2* and *Cx3cr1* expression in alveolar macrophages and neutrophils. (A) Representative contour plots of neutrophils and AMs from the lungs of mice infected with Mtb for 4 weeks. (B-C) *Ccr2*$^{RFP/+}$; *Cx3cr1*$^{GFP/+}$ mice were infected with Mtb for the indicated times, then MFIs of RFP and GFP in lung neutrophils and AMs (B) and MLN neutrophils (C) were measured by flow cytometry. All results presented as mean + SD and are derived from 1 experiment with 4 mice per genotype. Significance assessed by two-way (B) or one-way (C) ANOVA with multiple comparisons.

Supplemental Figure 3

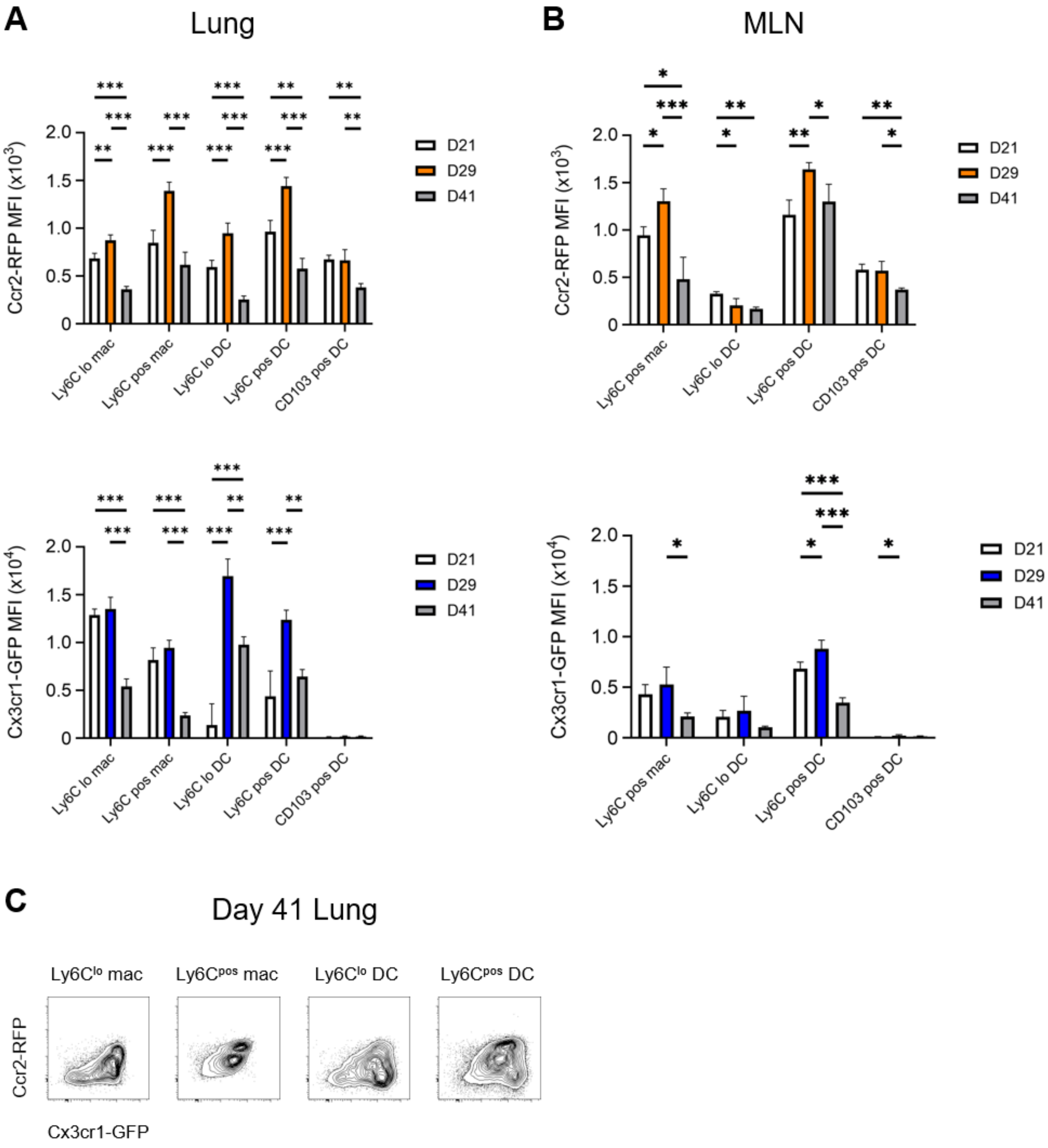

Supplemental Figure 3: *Ccr2* and *Cx3cr1* expression in lung and MLN phagocytes over time. *Ccr2*$^{RFP/+}$; *Cx3cr1*$^{GFP/+}$ mice were infected with Mtb for the indicated times, then lung (A) and MLN (B) cells were analyzed by flow cytometry. (C) Representative contour plots of the indicated populations from the lungs of mice infected for 41 days. All results presented as mean + SD and are derived from 1 experiment with 4 mice per genotype. Significance assessed by one-way ANOVA with multiple comparisons.

Supplemental Figure 4

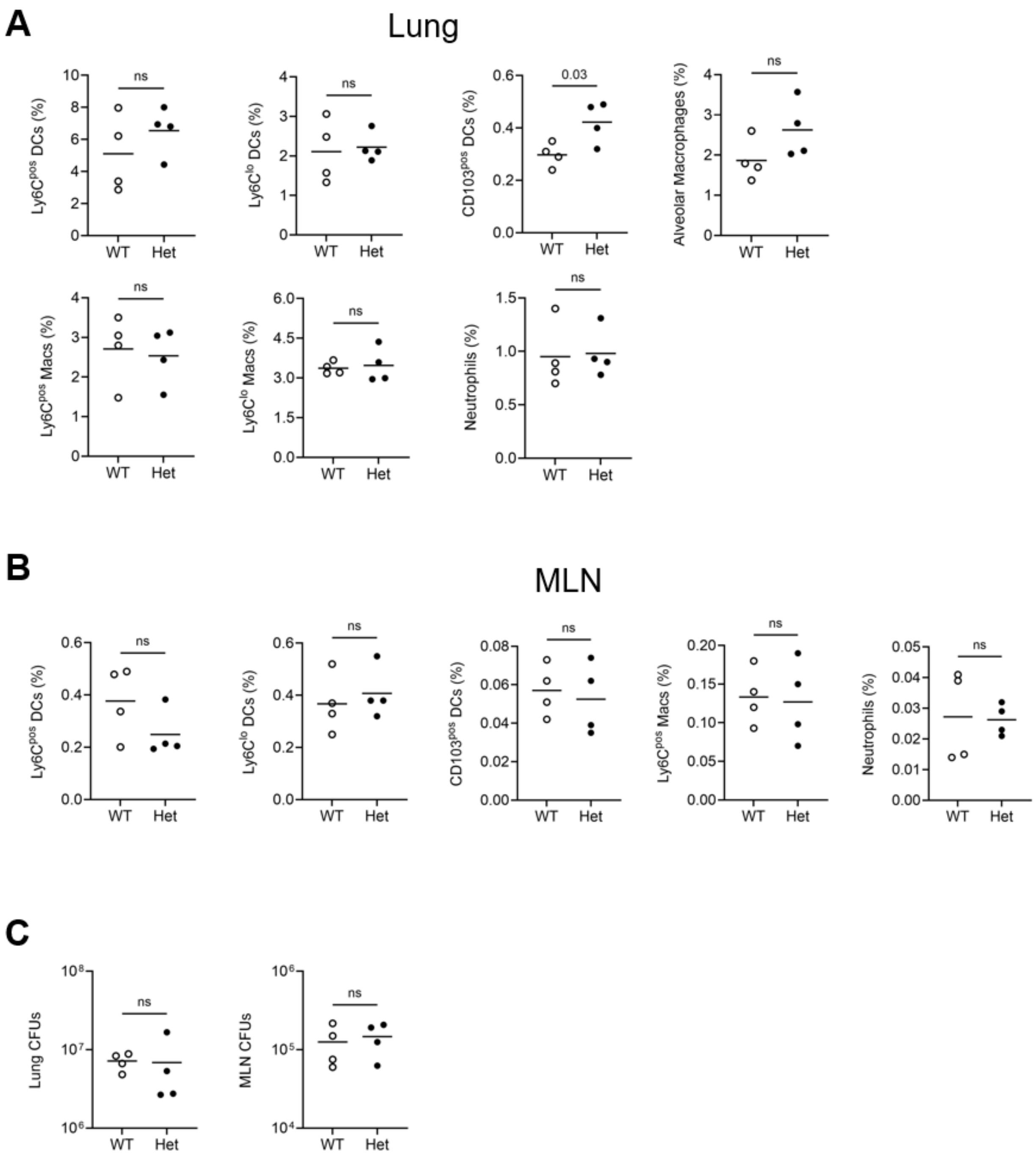

Supplemental Figure 4: Comparison of Mtb-infected *Ccr2*$^{+/+}$; *Cx3cr1*$^{+/+}$ and *Ccr2*$^{RFP/+}$; *Cx3cr1*$^{GFP/+}$ mice. Mice were infected with Mtb for 4 weeks, then lungs and MLNs were harvested. (A-B) Frequencies of the indicated lung (A) and MLN (B) populations among live cells by flow cytometry. (C) Quantitation of Mtb CFUs in lungs and MLNs. Data are from 1 of 2 experiments with similar results, each conducted with 4 mice per genotype. Horizontal bar represents the mean in all graphs. Significance assessed by *t* test.

Supplemental Figure 5

A

Lung

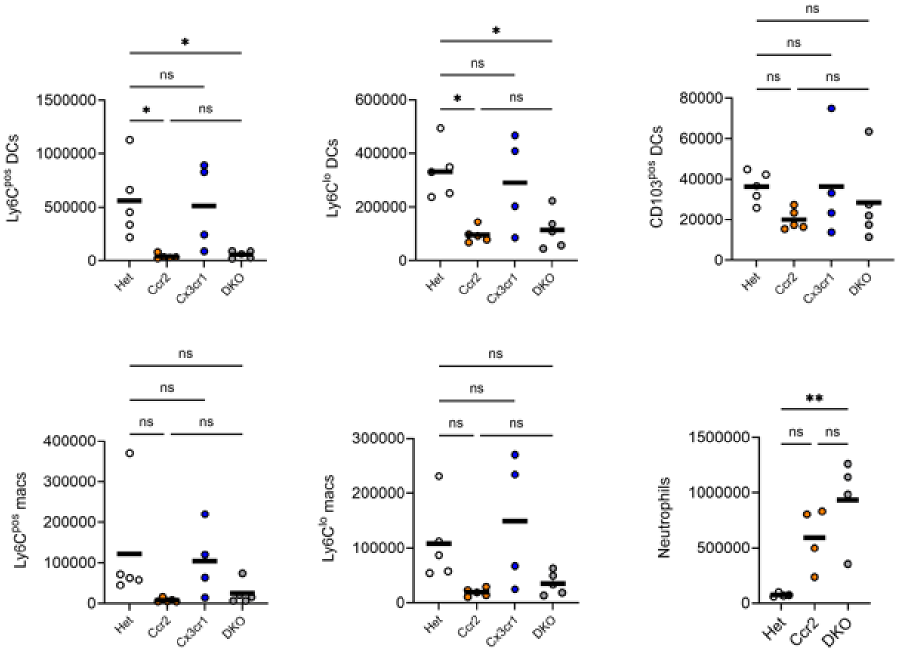

B

MLN

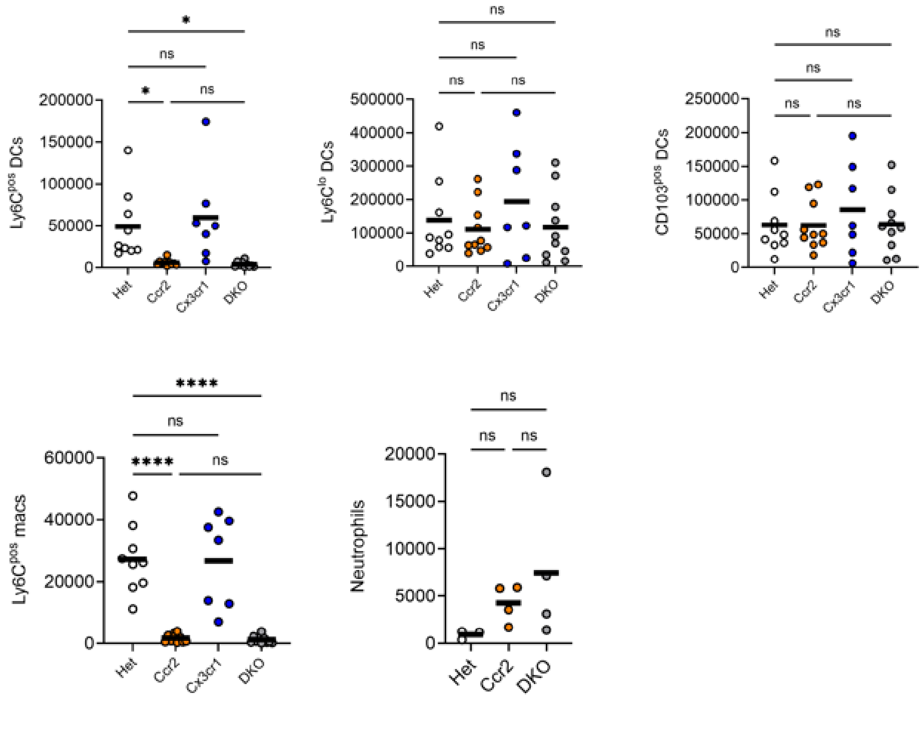

Supplemental Figure 5: Absolute phagocyte counts in the lungs and MLNs of CCR2-deficient, CX3CR1-deficient, and double knockout mice. Mice were infected, as in Fig. 2, and lung (A) and MLN (B) phagocytes were quantitated by flow cytometry. Data are from 1 of 2 experiments with similar results, each conducted with 4 mice per genotype. Horizontal bar represents the arithmetic mean in all graphs. Significance assessed by one-way ANOVA with multiple comparisons.

Supplemental Figure 6

A

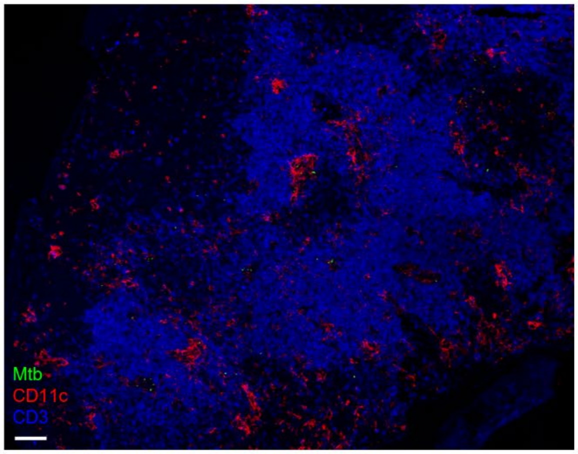

B

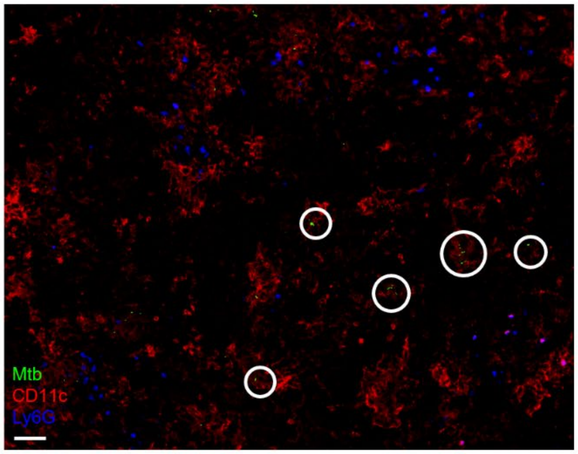

Supplemental Figure 6: Positioning of neutrophils and T cells in MLNs of infected double knockout mice. DKO mice were infected for 4 weeks with YFP-expressing Mtb, then MLNs were sectioned and stained by immunofluorescence for CD11c and either CD3 (A) or Ly6G (B). Circles in (B) denote areas of CD11c and YFP co-localization without adjacent Ly6G staining. Images are representative of 2 experiments.
